# End-to-end testing for stereotactic radiotherapy including the development of a Multi-Modality phantom

**DOI:** 10.1016/j.zemedi.2022.11.006

**Published:** 2022-12-18

**Authors:** Maya Shariff, Johanna Grigo, Siti Masitho, Tobias Brandt, Alexander Weiss, Ulrike Lambrecht, Willi Stillkrieg, Michael Lotter, Florian Putz, Rainer Fietkau, Christoph Bert

**Affiliations:** Department of Radiation Oncology, Universitätsklinikum Erlangen, Friedrich-Alexander-Universität Erlangen-Nürnberg (FAU), Erlangen, Germany; Comprehensive Cancer Center Erlangen-EMN (CCC ER-EMN), Erlangen, Germany

**Keywords:** End-to-End testing, SRS, SBRT, Quality Assurance, MRI distortion, Dynamic Tumor Tracking

## Abstract

****Purpose**:**

A new insert for a commercially available end-to-end test phantom was designed and in-house manufactured by 3D printing. Subsequently, the insert was tested for different stereotactic radiation therapy workflows (SRS, SBRT, FSRT, and Multimet) also in comparison to the original insert.

****Material and methods**:**

Workflows contained imaging (MR, CT), treatment planning, positioning, and irradiation. Positioning accuracy was evaluated for non-coplanar x-ray, kV- and MV-CBCT systems, as well as surface guided radiation therapy. Dosimetric accuracy of the irradiation was measured with an ionization chamber at four different linear accelerators including dynamic tumor tracking for SBRT.

****Results**:**

CT parameters of the insert were within the specification. For MR images, the new insert allowed quantitative analysis of the MR distortion. Positioning accuracy of the phantom with the new insert using the imaging systems of the different linacs was < 1 mm/degree also for MV-CBCT and a non-coplanar imaging system which caused > 3 mm deviation with the original insert. Deviation of point dose values was < 3% for SRS, FSRT, and SBRT for both inserts. For the Multimet plans deviations exceeded 10% because the ionization chamber was not positioned in each metastasis, but in the center of phantom and treatment plan.

****Conclusion**:**

The in-house manufactured insert performed well in all steps of four stereotactic treatment end-to-end tests. Advantages over the commercially available alternative were seen for quantitative analysis of deformation correction in MR images, applicability for non-coplanar x-ray imaging, and dynamic tumor tracking.

## Introduction

Radiotherapy is based on a meticulous workflow containing imaging, treatment planning, positioning at the linear accelerator, and the irradiation itself. Each step of the workflow must be performed carefully and within accuracy limits to ensure treatment success. Highly accurate workflows are in particular important for stereotactic treatments which are divided into stereotactic radiosurgery (SRS), fractionated stereotactic radiotherapy (FSRT) for intracranial tumors including multiple metastases (Multimet) and stereotactic body radiotherapy (SBRT) for extracranial tumors. The level of accuracy is recommended by AAPM TG 101 [1] or in the consensus statement from the DEGRO/DGMP Working Group for Stereotactic Radiotherapy and Radiosurgery [2], [3].

The accuracy of such workflows can be measured in end-to-end tests which quantify both, dosimetric and geometric accuracy. In those tests a phantom is treated like a patient and thus passed through each workflow step. Typical limits for geometric accuracy are < 1 mm (SRS), < 1.25 mm (FSRT and SBRT), and < 1.50 mm for SBRT at 3% dosimetric accuracy level [2], [3]. Numerous phantoms have been constructed for this or similar purposes [4], [5], [6], [7], [8], [9] including commercially available ones. With the variety of imaging, patient positioning, and treatment options currently available on the market, a single phantom solution hardly addresses all needs. One versatile phantom available since 2020 is RUBY [4], [10] (PTW, Freiburg, Germany) with multiple inserts addressing different needs. Brodbek et al. [5] have shown that non-coplanar imaging can be performed with the Mulitmet insert (PTW, Freiburg). Nevertheless, this insert is not suitable for MR imaging. Bohoudi [6], Mertens [7], Liu [8] and Gallas [9] all show different phantoms suitable for end-to-end testing. However, none of these phantoms combines the characteristics necessary for non-coplanar imaging, application of distortion correction in MR imaging, and dynamic tumor tracking (DTT) [11], [12]. Preliminary in-house investigations with the commercially available end-to-end test insert (SYSTEM QA) have shown that especially for MRI, DTT, MV-CBCT and non-coplanar kV imaging shortcomings exist which are likely caused by parallel bars and too little material variability embedded in the insert.

Consequently, within the scope of this manuscript, a new Multi-Modality QA insert was developed for the RUBY Phantom addressing the needs of the various stereotactic treatment workflows. Insert design and its performance are presented in comparison to the commercially available SYSTEM QA insert for four different stereotactic treatment workflows.

## Material and methods

### Phantom properties

**Figure 1:**
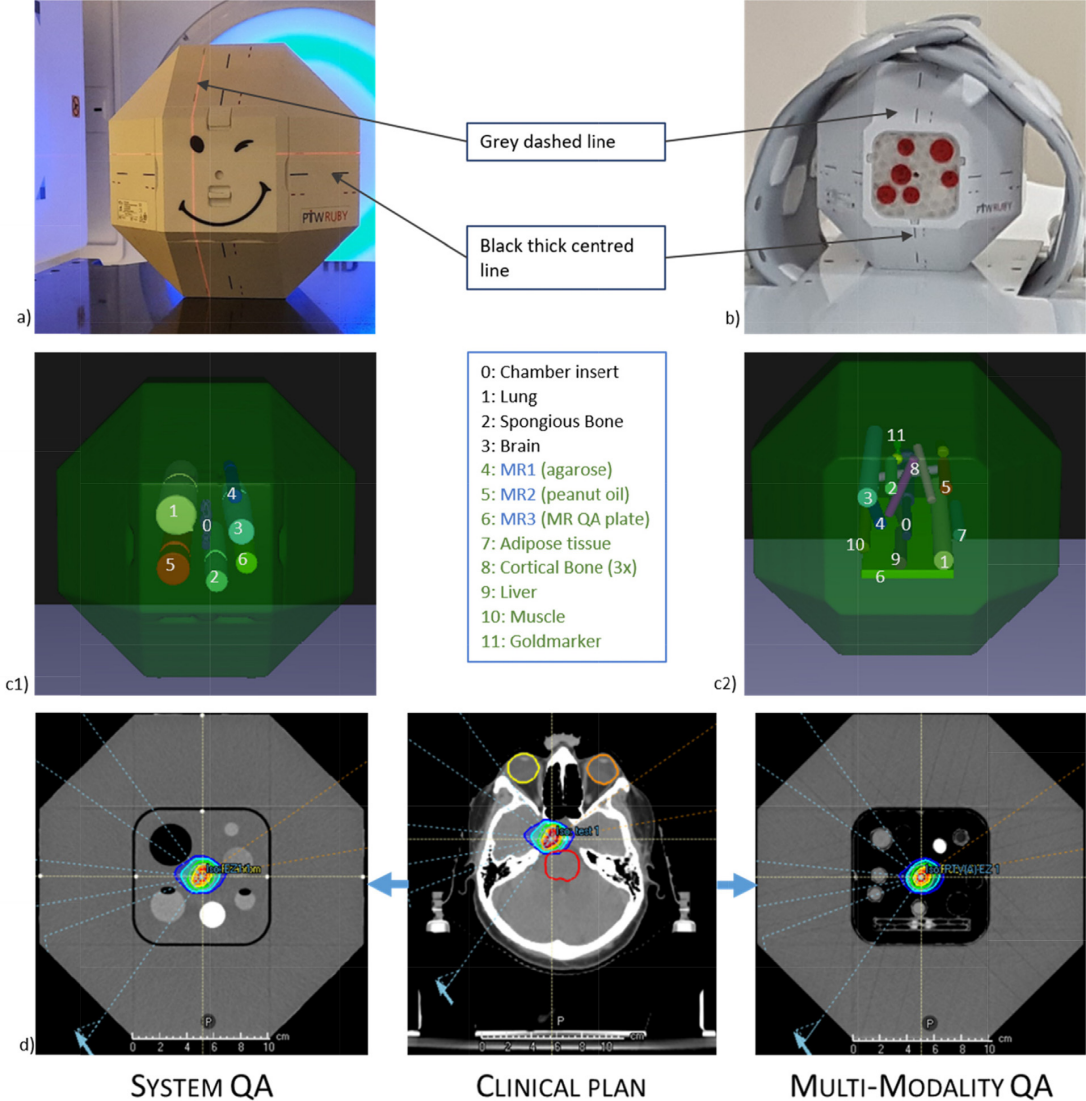
a) RUBY Phantom with the System QA Insert at VersaHD with 3 MR- and 3 CT-visible bars positioned according to the grey dashed lines [10]. b) RUBY Phantom at the MR Sola with the 18-channel Ultraflex coils (Siemens Healthineers, Erlangen, Germany) with the in-house made Multi-Modality QA insert with 2 MR-visible bars, 1 MR QA plate with 21 × 7 silicone spheres, 13 CT-visible bars and 1 silicone ball with an implanted gold marker. c1 + c2) Graphic representation of both inserts from the treatment planning system (TPS) Raystation. c1 shows the materials of the System QA insert and c2 shows the materials of the Multi-Modality QA insert. The numbering is described in the middle. Black: material in both inserts. Blue: System QA only. Green: Multi-Modality QA insert only. d) The clinical plans were transferred to both phantoms via Raystation. The isocenter of the plan was set to the geometrical center of the phantom.

RUBY [10] consists of a base made of polystyrene that allows insertion of different inserts (see Fig. 1).

The System QA (Fig. 1a) insert (PTW, Freiburg, Germany) contains three tubes filled with an MRI visible liquid and three additional tubes with lung, bone, and brain equivalent materials [4]. A new Multi-Modality QA insert (Fig. 1b) was designed within the scope of this work in RayStation (10B, RaySearch Laboratories, Stockholm, Sweden) and PrusaSlicer (Prusa, Prague, Czech Republic) and printed in-house with the 3D printer Original Prusa i3 MK3S+ (Prusa, Prague, Czech Republic). The Multi-Modality QA insert contains two MR visible tubes filled with agarose and peanut oil as reported by [13], [14] and an MR QA plate with 21 × 7 silicone spheres (diameter: 5 mm). In addition, a cylindrical gold marker (VISICOIL markers, IBA, Louvain-La-Neuve, Belgium) (20 mm length, 0.75 mm diameter) was implanted in a silicone ball, see Fig. 1c2) for QA of DTT. The soft silicone Duosil H (Shera, Werkstoff Technologie, Lemförde, Germany) with Shore hardness A 17 was used for the silicone components. As CT and x-ray visible structures, cylinders made of CT tissue equivalent material (lung, brain, cortical bone (3×), muscle (2×), spongious bone (2×), liver (2×) and adipose tissue (2×), all manufactured by QRM, Möhrendorf, Germany) were inserted. An overview of the materials and the dimensions of the individual components can be seen in Table A1 in the Supplementary material. With both inserts (System QA and Multi-modality QA) a point dose can be measured by inserting a ionization chamber (PinPoint 3D type 31022, PTW Freiburg, Germany) in the center of the phantom, see Fig. 1d. The chamber was used in combination with an UNIDOS electrometer (PTW, Freiburg, Germany). Region of interest (ROI) properties obtained with RayStation provide mean HU and electron density information using the hounsfield unit lookup table (HULUT) of the clinical workflow. These data were compared with the nominal values provided by the respective manufacturers of the inserts (see Table A1 in the Supplementary material).

In addition, the MR QA plate of the Multi-modality QA insert provides basic quantification of MR distortion with the rationale of checking if distortion correction was correctly applied to images used for treatment planning. For quantification, MR and CT images were registered manually. Afterwards, a self-written algorithm (Matlab 2021b, The MathWorks, Inc., Massachusetts, USA) detected the fiducials and calculated the gradient-dependent imaging fidelity using the distance from the middle of each fiducial in CT and MRI.

### Imaging

MR imaging of the phantom was performed with a 1.5T MAGNETOM Sola (Siemens Healthineers, Erlangen, Germany) using clinically performed sequences for images of stereotactic brain patients. To check the MR QA plate, the T1-weighted turbo-spin-echo-sequence (SPACE, TE = 19 ms, TR = 700 ms, BW = 399 Hz/pixel, resolution 0.5 × 0.5 × 1 mm^3^) was applied with 2D, 3D, and no distortion (ND) correction, see [15].

CT imaging was performed at a SOMATOM go.Open Pro (Siemens Healthineers, Erlangen, Germany) scanner using 120 kV, convolution kernel Hr40 and reconstructing 1 mm slices. The iterative metal artefact reduction (iMAR) correction was used.

### Workflows and treatment planning

**Figure 2:**
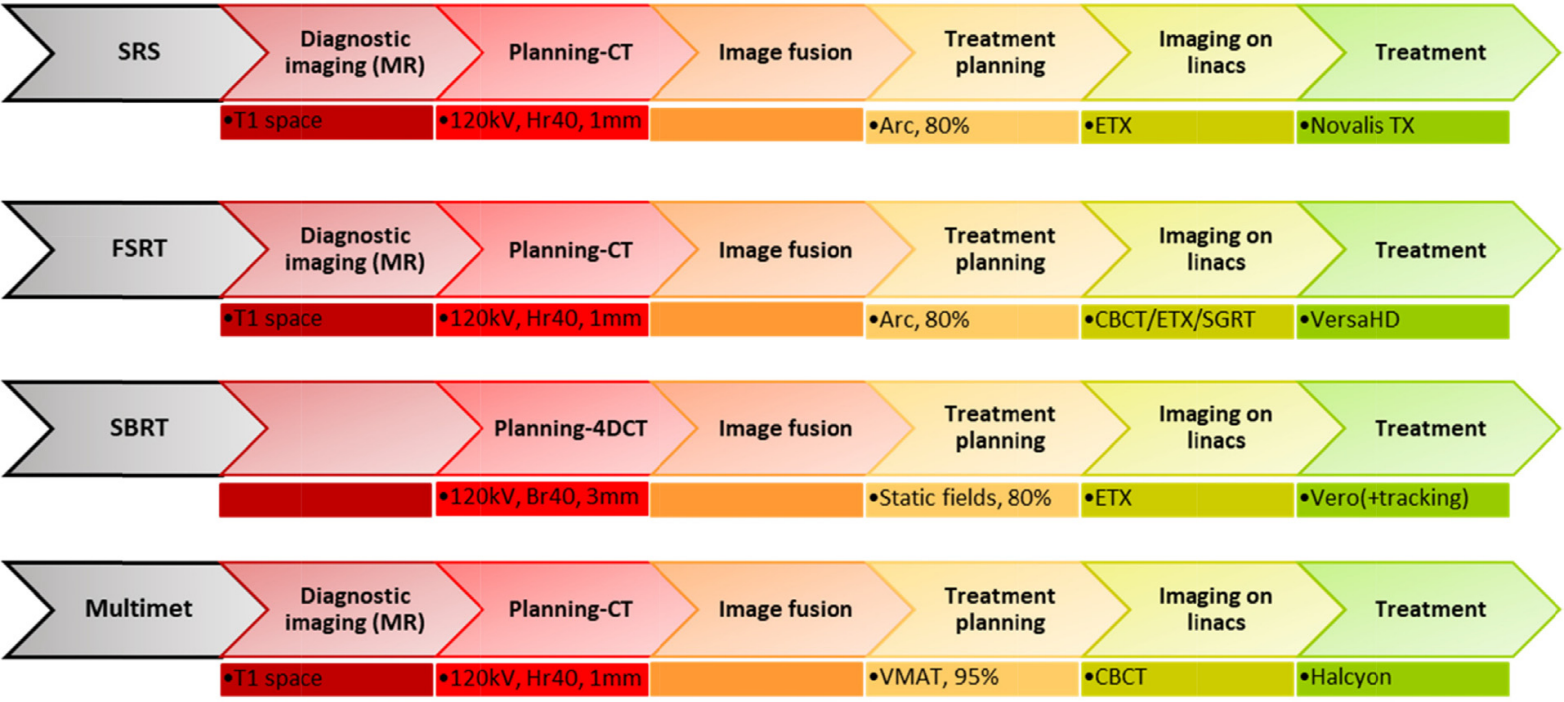
Schematic representation of the four end-to-end test workflows. Different imaging systems like Exactrac (ETX, Brainlab, Munich, Germany), cone beam CT (CBCT), surface-guided radiotherapy (SGRT) were applied.

The end-to-end test was analyzed as graphically summarized in Fig. 2. Ten clinical plans (details: Supplementary materials, Table A2) were taken from each of the workflows SRS, FSRT, SBRT including Tracking, Multimet, which were calculated on the CT of the respective phantoms (System QA and Multi-modality QA). MRI/CT image fusion and treatment planning were performed in RayStation with a 0.15 × 0.15 × 0.15 cm^3^ dose grid and the collapsed cone calculation algorithm for each plan.

In brief (details provided in the Supplementary materials), SRS is applied for individual brain metastases to deliver 18–20 Gy to the 80% isodose using 2–4 static arcs with different table rotations. In case of larger targets (2–4 cm), FSRT is used to apply 12 × 4 Gy [15], [16], [17], [18] to the 80% isodose using static arcs or VMAT techniques while > 3 brain metastases (Multimet) are treated VMAT-based with 10 × 3 Gy normalized to the 95% isodose. In case of lung or liver lesions, SBRT (12 × 6 Gy, 60% or 80% isodose) was applied in expiration or based on DTT. DTT is based on a correlation motion of external surrogate and internal gold marker position established at the beginning of each treatment fraction. During delivery external surrogate data is processed by the model to predict the internal tumor position which is used to compensate during beam delivery by adjusting a gimbaled linac head [11], [12], [19].

### Evaluation of image guidance

To analyze the feasibility of both inserts for end-to-end testing of each positioning system and to determine the positioning accuracy, three imaging types were performed on linacs with all imaging systems (non-coplanar kV-x-ray (ExacTrac, Brainlab, Munich), SGRT (AlignRT, VisionRT, London, UK), kV- and MV-CBCT) and both inserts.

For each imaging measurement, the base body with the corresponding insert was positioned on the treatment couch using the laser system. For this purpose, the phantom’s surface contains black lines indicating the center of the phantom base and grey lines that are used to position the phantom with a defined offset of 14 mm (coronal), 18 mm (transversal), 25 mm (sagittal), see Fig. 1a. An image was acquired with the respective imaging system. A displacement vector was then determined with the respective software and the reference image, whereupon the couch was displaced by these values. The test assumes that the kV-MV isocenter of each of the linacs matches perfectly, i.e. that the determined displacement vector corresponds to the offset between grey and black lines.

### Dosimetric evaluation

The 10 treatment plans of each workflow were applied at the corresponding linac and the point dose was measured with a PinPoint ionization chamber positioned isocentrically. The measured dose values were corrected according to DIN 6809-8 [20], depending on field size, and compared with the calculated point dose from the TPS.

In accordance with [21], all ten Vero plans were additionally irradiated in the presence of motion. For this purpose, the base phantom with the corresponding insert was aligned on a 1D motion platform using the laser system (2 cm PMMA, 5 cm air between PMMA and couch-top, see Fig. A1 in Supplementary material) moving in in-plane direction (sinus, peak-to-peak A = 10 cm, T = 4 s). For DTT, a gold marker, which can be seen in the fluoroscopic images, is implemented in the Multi-Modality QA insert. DTT was not feasible with the System QA insert which does not contain a gold marker.

## Results

### Evaluation of insert properties

Table A1 in the Supplementary material lists the nominal and determined values of HU and density of all structures embedded in the inserts. These are within the specification values for both inserts.

**Figure 3:**
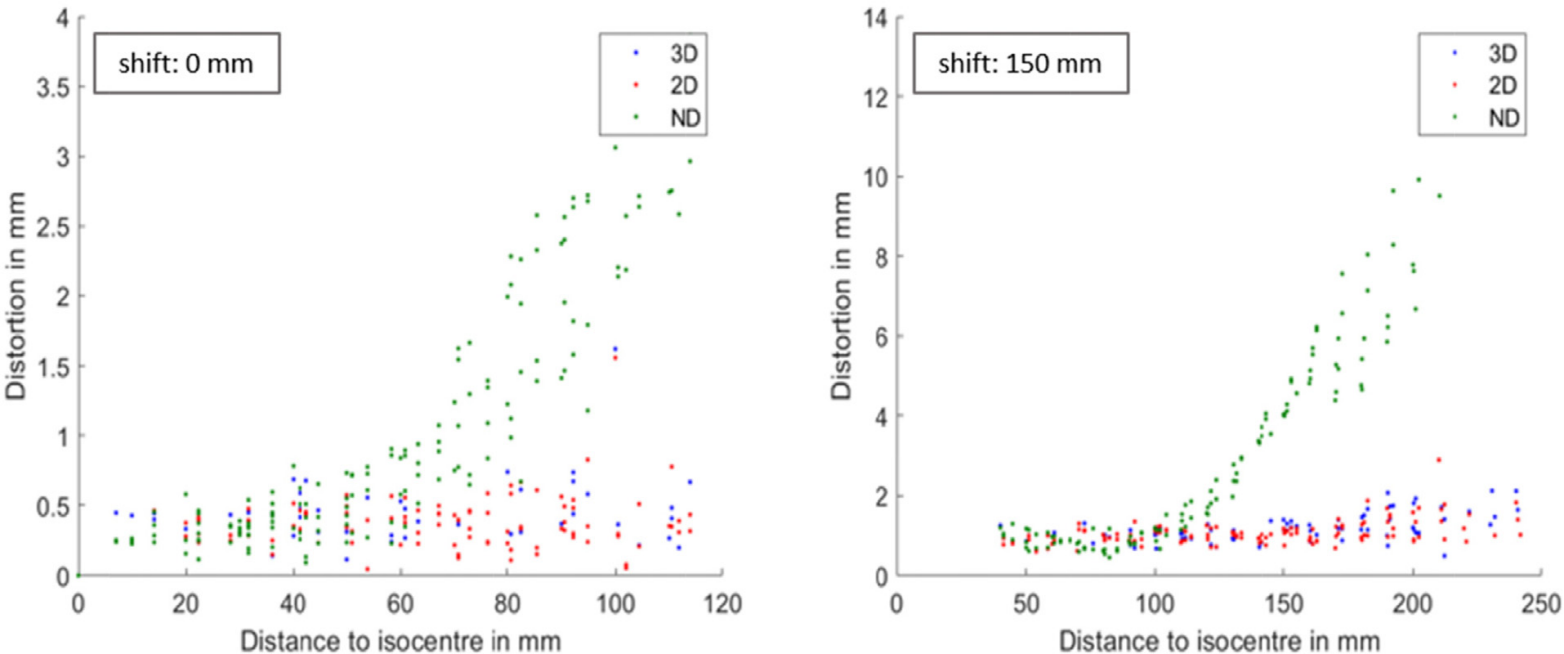
Distortions of points of a correctly (left) and incorrectly (right, shift of 150 mm in head direction) positioned phantom relative to their distance from isocenter. Please note the different scale of the axes. ND: no distortion correction.

Regarding MR-imaging both inserts allowed to judge qualitatively if 3D distortion correction was activated (see Fig. A2, Supplementary material). For the Multi-modality QA insert, also quantitative evaluation of the distortions was possible, due to the MR QA plate with the silicone spheres included in this new insert. Fig. 3 shows that 2D/3D distortion correction minimize distortions in the brain region to < 1 mm for a phantom positioned in the isocenter and to < 2 mm in case of a 150 mm shift. In the shifted position scans without distortion correction lead to distortions > 2 mm at distances > 100 mm from isocenter.

### Evaluation of image guidance

Table 1 shows the imaging performance results of both inserts for the 8 different imaging systems. Imaging of both inserts using kV-CBCT (Versa, Halcyon, and Vero) and SGRT (AlignRT) worked well. However, there were problems associated with imaging of the Exactrac and MV-CBCT. Due to their small field-of-view (FoV) and the parallel rods of the System QA insert the phantom’s position in the longitudinal direction could not be determined correctly. In the Multi-modality QA insert, the limited FoV was already considered during the development. The bars were arranged within the insert slightly oblique. As a result, accurate automatic positioning was accurately possible with all imaging systems.

**Table 1:** Mean absolute deviations between expected and measured offset for phantom positioning on all linacs. Green: values between −0.5 mm and +0.5 mm; yellow: values between 0.5 mm and 1.0 mm; red: values > 1.0 mm.

### Dosimetric evaluation

**Figure 4:**
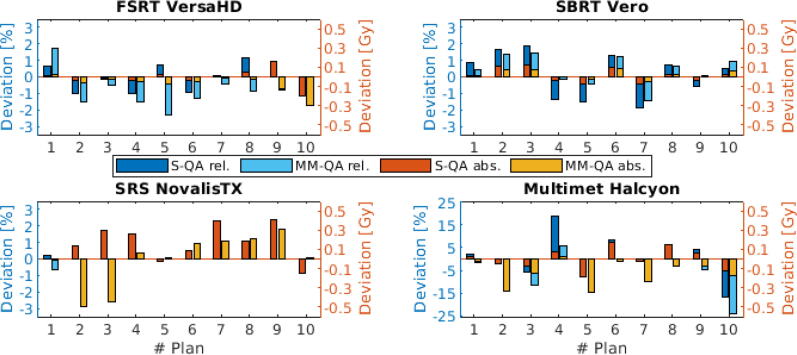
Verification of the point dose values. On the left (blue) ordinate the differences to the target dose are plotted in percentage and on the right (orange) ordinate in absolute Gy. All measurements were performed with System QA (S-QA) and Multi-Modality QA (MM-QA). At each linac, 10 plans per insert were measured.

Fig. 4 shows the results for each of the 10 treatment plans of each treatment workflow. The mean values of three measurements each were used. The mean deviations of the absolute point dose measurements for the workflows SRS, FSRT, SBRT, and Multimet were (0.16 ± 0.17) Gy, (−0.01 ± 0.08) Gy, (0.01 ± 0.07) Gy and (−0.00 ± 0.11) Gy, respectively for Multi-modality QA insert. The mean deviations for these workflows were (−0.00 ± 0.25) Gy, (−0.07 ± 0.08) Gy, (0.03 ± 0.04) Gy and (−0.13 ± 0.12) Gy, respectively for System QA insert. For SBRT, mean dosimetric deviation of DTT compared to the application without phantom motion changed from (−0.03 ± 0.11) Gy to (−0.02 ± 0.04) Gy. In the Multimet workflow, the relative deviation was (6.85 ± 7.88) % for System QA insert and (−0.22 ± 8.87) % for Multi-modality QA insert.

The measured dose deviations between both inserts showed no difference at p = 0.01 significance level when analyzed only for the 30 treatment plans based on SRS, FSRT, and SBRT (t-test for dependent means, *p *= 0.04). When including the Multimet measurements, deviations of > 10 % at small absolute deviations of < 0.5 Gy were observed and mean deviations were significantly different (*p *= 0.002). Since only one isocentric point dose was recorded per Multimet plan, only out-of-target dose could be detected at the chamber position.

## Discussion

A check of the radiotherapy chain must be carried out at regular intervals. In this process, the same workflow as for the patients is followed with a dedicated phantom. Here, this test was applied to the workflows of four stereotactic treatment options using a newly designed and in-house manufactured Multi-Modality QA insert for the RUBY phantom in comparison to the commercially available System QA insert.

Concerning phantom insert properties the specifications were met for CT and MR imaging. Quantitative evaluation of distortion correction was possible by the integrated MR QA plate. However, the limitations of an in-house fabrication (roundness and homogeneity) of the spheres resulted in unreliable detection of distortions below 0.5 mm (CT and MR were performed with a slice thickness of 1 mm; silicone balls have a diameter of 5 mm). Despite that drawback, distortion data are comparable to measurements conducted at the identical MR scanner with the commercially available MR CIRS Phantom (Model 604-GS, Sun Nuclear, Norfolk, USA) [22], and also to the monthly QA measurements using the THETIS 3D MR Distortion Phantom (LAP GmbH, Lüneburg, Germany) [23]. Long term stability of the MR QA plate is not yet available. However, mechanical stability is expected since silicon and not gel (suszeptible to evaporation) was used to produce the MR signal.

For image guidance, the Multi-Modality QA insert showed advantages for ExacTrac and MV-CBCT at comparable outcome < ± 0.5 mm to the System QA insert for the other imaging modalities. Automatic positioning using the System QA insert was particularly difficult for the Exactrac (4.2 mm) and MV-CBCT (6.4 mm) and significantly exceeded the recommended values of [3]. This behavior can be attributed to the non-parallel CT-visible rods that allow image guidance also for small FOV modalities. In principle, precise positioning of the phantom can also be achieved with the commercially available System QA insert of RUBY but requiring manual fusion rather than the automatic procedure used in daily treatments. Inserts like the LINAC QA are not compatible for end-to-end testing due to missing dose measurement capability. The nominal values for the motionless SBRT, FSRT and Mulitmet with kV-CBCT were fulfilled with both inserts (System QA: < 0.3 mm, Multi-modality QA: 0.2 mm).

Apart from geometric accuracy, the recommendations reported in [3] further address dosimetric accuracy with point-based plan-to-measurement differences of at most 3 % for target volumes ≥ 2 cm^3^ in homogeneous phantoms. Dosimetrically, the new insert was comparable to the commercially available alternative for SRS and FSRT. With respect to the Multimet plans, dosimetric accuracy was difficult to reach with both inserts (> 20% relative deviation, each), since measurements were not possible simultaneously in the center of multiple metastases. Absolute deviations of < 0.5 Gy were comparable to the other workflows but the insert could potentially be improved by allowing multiple ionization chambers in parallel as reported already [4], [5]. An advantage of the Multi-Modality QA insert is its applicability for DTT due to the embedded fiducial.

Several papers reporting on end-to-end tests have already been published. Some of the groups have also built or printed a phantom themselves and tested its feasibility, others report on tests using commercially available solutions. Representative examples are Kry et al. [24] (0.9 ± 0.15 mm displacement between imaging and radiation isocenter) and Mertens et al. [7] (mean dose deviation of 1.82% ± 1.03%), who each validated their results obtained with the in-house developed phantom with commercial phantoms. Both groups focused on the feasibility of the Winston-Lutz test, which is not addressed in this paper. Similarly, Poppinga et al. [4] started by comparing the RUBY Phantom with the different inserts to phantoms previously available on the market [4]. As in our data (Table 1) they report offset deviations < 1 mm for CBCT based imaging for all systems and also dosimetric deviations of < 3% agree with our findings. Zakjevskii et al. [25] developed an end-to-end test for head and neck IMRT treatments with a focus on dosimetric accuracy. Measurements were performed with film, ionization chambers, and optically stimulated luminescent dosimeters. Ionization chamber data (< 3% deviation to expectation) were comparable to the Multi-Modality QA insert application reported here. Film measurements would be beneficial for the Multi-Modality QA insert e.g. to address the shortcoming found in Multimet dosimetry and might be addressed in future iterations of the insert. Niebuhr et al. and Hoffmans et al. [13], [26] have created a pelvic phantom suitable for end-to-end testing of MR-guided radiotherapy workflows. Based on their investigations, the idea of adding peanut oil to achieve an MR-visible bar was adopted in the Multi-Modality QA insert. Apart from that, the approaches differ since their study mainly addressed the accuracy of plan adaptations in case of phantom deformation within an adaptive MR-guided radiation therapy (MRgRT) workflow. Brodbek et al. [5] evaluated the RUBY with the MultiMet insert (PTW, Freiburg, Germany) in a head phantom. This insert allows for 3 ionization chamber positions in the phantom (isocentric, peripheral top and bottom). Ionization chamber measurements thus required dedicated rather than clinical treatment plans for that special geometry. Deviations of up to 6.9 % occurred in their point dose measurements, comparable to the Multi-Modality QA insert.

## Conclusion

A new Multi-Modality QA insert for RUBY-based end-to-end testing was designed, manufactured, and successfully tested for numerous treatment workflows based on MRT and CT, treatment planning, and 4 different linear accelerators with 8 different imaging modalities. The insert allows quantification of MR distortions, dynamic tumor tracking, and check of HU. Minor disadvantages result from the single bore for an ionization chamber which results in larger dosimetric deviation in case of a multiple metastases treatment workflow.

## Declaration of Competing Interest

The authors declare that they have no known competing financial interests or personal relationships that could have appeared to influence the work reported in this paper.
