# Supplementary material for: End-to-end testing for stereotactic radiotherapy including the development of a Multi-Modality phantom
Source: Z Med Phys. 2022 Dec 18;34(3):477–84. doi: 10.1016/j.zemedi.2022.11.006 (PMC11384089; doi:10.1016/j.zemedi.2022.11.006)
Supplement: Supplementary data 1 [file mmc1.docx]

# Supplementary material

Figure A1: MULTI-MODALITY QA insert on the Vero on the moving plate for markerless dynamic tumor tracking.

Figure A2: Comparison between the different QA inserts for 3D distortion and non-distortion (ND) corrected MRI images (orange) registered with the CT (blue).

Table A1: Phantom properties. Materials of Multi-Modality QA insert, its HU and density values, each nominal and measured values. System QA insert has also lung, brain and cortical bone bars und has the same values.

| Multi-Modality QA |  |  | **CT values [HU]** | | | | **Density [g/cm³]** | | | | |
| --- | --- | --- | --- | --- | --- | --- | --- | --- | --- | --- | --- |
|  | Diameter [cm] | Length [cm] | nominal | measured | Δ_relative [%]_ | Δ_absolute_ | nominal | measured | Δ_relative [%]_ | Δ_absolute_ |  |
|  |  |  |  |  |  |  |  |  |  |  |  |
| ***Lung*** | 1.5 | 22.5 | -740 | -752 | -4.6 | -12 | 0.26 | 0.23 | -11.5 | -0.03 |  |
| ***Brain*** | 1.5 | 22.5 | 39 | 40 | 0.1 | 1 | 1.04 | 1.07 | 2.8 | 0.03 |  |
| ***Cortical Bone*** | 0.8 | 0.6 | 1257 | 1141 | -5.1 | -116 | 1.68 | 1.65 | -1.7 | -0.03 |  |
| ***Muscle*** | 1.0 | 0.85 | 44 | 36 | -0.7 | -8 | 1.05 | 1.06 | 0.9 | 0.01 |  |
| ***Spongious Bone*** | 1.0 | 0.85 | 325 | 260 | -4.9 | -65 | 1.18 | 1.21 | 2.5 | 0.03 |  |
| ***Liver*** | 1.0 | 0.85 | 55 | 36 | -1.8 | -19 | 1.06 | 1.08 | 1.9 | 0.02 |  |
| ***Adipose tissue*** | 1.0 | 0.85 | -25 | -84 | -6.1 | -59 | 0.95 | 0.95 | 0.0 | 0.00 |  |
| ***MR1 (agarose)*** | 1.0 | 0.85 |  | 5 |  |  |  | 1.03 |  |  |  |
| ***MR2 (peanut oil)*** | 1.0 | 0.85 |  | -125 |  |  |  | 0.90 |  |  |  |
| ***MR QA*** |  | 7x20x0.8 |  | 130 |  |  |  | 1.13 |  |  |  |

## Treatment plan and technique details

For SRS of individual brain metastases, the Novalis Tx Varian Medical Systems, Inc., Palo Alto, CA, USA and Brainlab AG, Munich, Germany; 2.5 mm MLC leaves) was used to deliver 18 or 20 Gy prescribed on the 80 % isodose relative to the reference point in the target volume enclosing the PTV in a single session [1-3]. All SRS plans were planned with a Static Arc (rotational therapy where every segment has an identical aperture) and different table rotations.

If the individual brain metastases exceeded a certain size (2 to 4 cm diameter), they were irradiated in multiple fractions (FSRT). In compliance with the study protocol of the prospective multicenter FSRT-Trial, a fractionation scheme of 12 x 4 Gy was used [4-7]. The FSRT plans were planned with Static Arc or VMAT applied using a VersaHD linac (Elekta AB, Stockholm, Sweden). Also, for this treatment, the dose (12 x 4 Gy) was prescribed to the 80 % isodose encompassing the PTV.

SBRT was used for treatment of lesions in lung or liver which were irradiated in expiration (receiving breathing commands) using the Vero 4DRT system (Mitsubishi Heavy Industries, Ltd., Tokyo, Japan and Brainlab AG, Munich, Germany). The dose of 12 x 6 Gy was prescribed to the 60 % or 80 % isodose enclosing the PTV [8, 9]. Furthermore, liver metastases were irradiated using dynamic tumor tracking: based on a correlation motion of external surrogate and internal gold marker position established at the beginning of each treatment fraction, tumor motion was compensated during beam delivery [10]. The Vero uses a gimbaled linac head to follow the tumor motion during a dynamic tumor tracking (DTT) treatment [11, 12].

For treatment of fractionated multiple (> 3) brain metastases (Multimet) the Halcyon linac (Varian Medical Systems, Inc., Palo Alto, CA, USA) was used (fractionation: 10 x 3 Gy, normalized to 95 % isodose enclosing the PTV). Therefore, they were irradiated with the VMAT technique, as this means a shorter treatment time for the patient and, at the same time, the areas between the metastases could be spared comparable to single irradiation of all metastases [13].

Table A2: Properties of treatment plans

| Workflow | Linac | technique | # fields/arcs | couch/ring angle | total MU | normalization | Target volume | Prescribed dose | # metastases |
| --- | --- | --- | --- | --- | --- | --- | --- | --- | --- |
| FSRT (1) | Versa | VMAT | 2 | 0 | 642 | 80% | 32.22 | 12x 4Gy | 1 |
| FSRT (2) | Versa | VMAT | 2 | 0/30 | 666 | 80% | 11.82 | 12x 4Gy | 1 |
| FSRT (3) | Versa | VMAT | 2 | 0 | 1127 | 80% | 14.65 | 12x 4Gy | 1 |
| FSRT (4) | Versa | VMAT | 4 | 0/0/65/65 | 667 | 95% | 3.52 | 12x 4Gy | 1 |
| FSRT (5) | Versa | VMAT | 2 | 0/290 | 636 | 80% | 21.08 | 12x 4Gy | 1 |
| FSRT (6) | Versa | VMAT | 2 | 0/280 | 608 | 80% | 16.18 | 12x 4Gy | 1 |
| FSRT (7) | Versa | VMAT | 2 | 0/300 | 684 | 80% | 7.33 | 12x 4Gy | 1 |
| FSRT (8) | Versa | VMAT | 3 | 0 | 1207 | 80% | 11.86 | 12x 4Gy | 1 |
| FSRT (9) | Versa | Static Arc | 5 | 0/340/320/295/270 | 618 | 80% | 5.67 | 12x 4Gy | 1 |
| FSRT (10) | Versa | VMAT | 2 | 0 | 651 | 80% | 32.46 | 12x 4Gy | 1 |
| SRS (1) | Novalis Tx | Static Arc | 5 | 330/0/0/315/270 | 3752 | 80% | 1.80 | 1x 18Gy | 1 |
| SRS (2) | Novalis Tx | Static Arc | 4 | 315/270/0/45 | 4017 | 80% | 3.06 | 1x 20Gy | 1 |
| SRS (3) | Novalis Tx | Static Arc | 4 | 0/0/80/45 | 2943 | 80% | 1.37 | 1x 20Gy | 1 |
| SRS (4) | Novalis Tx | Static Arc | 4 | 0/25/50/75 | 3468 | 80% | 0.77 | 1x 20Gy | 1 |
| SRS (5) | Novalis Tx | Static Arc | 4 | 0/308/340/80 | 3382 | 80% | 0.54 | 1x 20Gy | 1 |
| SRS (6) | Novalis Tx | Static Arc | 4 | 0/0/320/70 | 3176 | 80% | 2.26 | 1x 20Gy | 1 |
| SRS (7) | Novalis Tx | Static Arc | 3 | 0/45/90 | 3355 | 80% | 1.62 | 1x 20Gy | 1 |
| SRS (8) | Novalis Tx | Static Arc | 5 | 0/0/40/320/270 | 4054 | 80% | 3.02 | 1x 20Gy | 1 |
| SRS (9) | Novalis Tx | Static Arc | 3 | 0/0/300 | 3662 | 80% | 0.38 | 1x 18Gy | 1 |
| SRS (10) | Novalis Tx | Static Arc | 5 | 0/0/45/90/315 | 3572 | 80% | 0.53/10.57 | 1x 20Gy + 1x18Gy | 1 |
| Multimet (1) | Halcyon | VMAT | 3 | 0 | 782 | 95% | 2.49 | 10x 4Gy | 4 |
| Multimet (2) | Halcyon | VMAT | 2 | 0 | 614 | 95% | 2.12 | 5x 3Gy | 5 |
| Multimet (3) | Halcyon | VMAT | 2 | 0 | 769 | 95% | 8.10 | 8x 3Gy | 7 |
| Multimet (4) | Halcyon | VMAT | 3 | 0 | 1135 | 95% | 4.69 | 12x 4Gy | 7 |
| Multimet (5) | Halcyon | VMAT | 4 | 0 | 1349 | 95% | 10.57 | 8x 3Gy | 11 |
| Multimet (6) | Halcyon | VMAT | 4 | 0 | 1058 | 95% | 28.09 | 5x 3Gy | 10 |
| Multimet (7) | Halcyon | VMAT | 4 | 0 | 1031 | 95% | 25.51 | 16x 3Gy | 8 |
| Multimet (8) | Halcyon | VMAT | 2 | 0 | 673 | 95% | 0.86 | 12x 4Gy | 2 |
| Multimet (9) | Halcyon | VMAT | 3 | 0 | 795 | 95% | 2.05 | 12x 4Gy | 2 |
| Multimet (10) | Halcyon | VMAT | 3 | 0 | 1038 | 95% | 8.12 | 7x 3Gy | 19 |
| SBRT (1) | Vero | 3DCRT | 8 | 10/0/0/0/15/0/15/15 | 895 | 80% | 25.74 | 12x 6Gy | 1 |
| SBRT (2) | Vero | 3DCRT | 6 | 0/0/15/0/15/0 | 955 | 80% | 6.26 | 12x 6Gy | 1 |
| SBRT (3) | Vero | Static Arc | 4 | 0/10/345/0 | 1452 | 80% | 6.72 | 12x 7Gy | 1 |
| SBRT (4) | Vero | SMLC | 6 | 350/350/10/10/10/0 | 918 | 60% | 10.,28 | 12x 6Gy | 1 |
| SBRT (5) | Vero | SMLC | 7 | 340/20/350/350/20/0/20 | 1187 | 80% | 23.45 | 12x 7Gy | 1 |
| SBRT (6) | Vero | 3DCRT | 7 | 0 | 816 | 80% | 55.98 | 12x 6Gy | 1 |
| SBRT (7) | Vero | 3DCRT | 8 | 350/20/20/0/0/0/0/0 | 836 | 80% | 21.96 | 12x 5Gy | 1 |
| SBRT (8) | Vero | 3DCRT | 6 | 0 | 668 | 95% | 16.59 | 10x 4Gy | 1 |
| SBRT (9) | Vero | 3DCRT | 7 | 0/0/0/15/15/15/10 | 1167 | 80% | 69.01 | 12x 6Gy | 1 |
| SBRT (10) | Vero | VMAT | 2 | 15/345 | 756 | 80% | 35.35 | 12x 6Gy | 1 |

# References

1. Lippitz, B., et al., *Stereotactic radiosurgery in the treatment of brain metastases: The current evidence.* Cancer Treatment Reviews, 2014. **40**(1): p. 48-59.

2. Hardcastle, N. and W.A. Tome, *On a single isocenter volumetric modulated arc therapy SRS planning technique for multiple brain metastases.* J Radiosurg SBRT, 2012. **2**(1): p. 1-9.

3. Soliman, H., et al., *Stereotactic radiosurgery (SRS) in the modern management of patients with brain metastases.* Oncotarget, 2016. **7**(11): p. 12318-30.

4. Oehlke, O., et al., *Amino-acid PET versus MRI guided re-irradiation in patients with recurrent glioblastoma multiforme (GLIAA) – protocol of a randomized phase II trial (NOA 10/ARO 2013-1).* BMC Cancer, 2016. **16**(1): p. 769.

5. Putz, F., et al., *FSRT vs. SRS in Brain Metastases-Differences in Local Control and Radiation Necrosis-A Volumetric Study.* Front Oncol, 2020. **10**: p. 559193.

6. Putz, F. and R. Fietkau, *FSRT-Trial: Efficacy and Safety of Fractionated Stereotactic Radiation Therapy versus Single Fraction Stereotactic Radiosurgery for Large Brain Metastases (AG-NRO-05)*. 2022.

7. Mack, A., et al., *Quality assurance in stereotactic space. A system test for verifying the accuracy of aim in radiosurgery.* Med Phys, 2002. **29**(4): p. 561-8.

8. Lubgan, D., et al., *12 × 6 Gy stereotactic radiotherapy for lung tumors. Is there a difference in response between lung metastases and primary bronchial carcinoma?* Strahlenther Onkol, 2022. **198**(2): p. 110-122.

9. Spindeldreier, C.K., et al., *MR-guided radiotherapy of moving targets.* Der Radiologe, 2021.

10. Witulla, B., et al., *On PTV definition for glioblastoma based on fiber tracking of diffusion tensor imaging data.* PLoS One, 2020. **15**(1): p. e0227146.

11. Ziegler, M., et al., *Performance of Makerless Tracking for Gimbaled Dynamic Tumor Tracking.* Z Med Phys, 2020. **30**(2): p. 96-103.

12. Ziegler, M., et al., *Performance of gimbal-based dynamic tumor tracking for treating liver carcinoma.* Radiat Oncol, 2018. **13**(1): p. 242.

13. Petoukhova, A., et al., *Quality of Automated Stereotactic Radiosurgery Plans in Patients with 4 to 10 Brain Metastases.* Cancers, 2021. **13**(14): p. 3458.
